# Challenges to implementation of developmental screening in urban primary care: a mixed methods study

**DOI:** 10.1186/1471-2431-14-16

**Published:** 2014-01-21

**Authors:** Deanna L Morelli, Susmita Pati, Anneliese Butler, Nathan J Blum, Marsha Gerdes, Jennifer Pinto-Martin, James P Guevara

**Affiliations:** 1 Policylab: Center to Bridge Research, Practice, & Policy, The Children’s Hospital of Philadelphia, 34th and Civic Center Boulevard, CHOP North, Room 1531, 3535 Market Street, Philadelphia, PA 19104, USA; 2 Department of Pediatrics, State University of New York at Stony Brook and Long Island Children’s Hospital, Health Sciences Center T11-020, Stony Brook, NY 11794, USA; 3 Philadelphia Veterans Medical Center, 3900 Woodland Avenue, Philadelphia, PA 19104, USA; 4 Division of Child development, Rehabilitation, and Metabolic Disease, Children’s Hospital of Philadelphia, 3550 Market Street, 3rd Floor, Philadelphia, PA 19104, USA; 5 School of Nursing, University of Pennsylvania, 418 Curie Boulevard, Philadelphia, PA 19104, USA; 6 Center for Clinical Epidemiology and Biostatistics, Perelman School of Medicine, University of Pennsylvania, 8th Floor, Blockley Hall, 423 Guardian Drive, Philadelphia, PA 19104, USA

**Keywords:** Child development, Primary care, Screening, Hospitals, Urban, Developmental assessment

## Abstract

**Background:**

Research is needed to identify challenges to developmental screening and strategies for screening in an urban pediatric setting.

**Methods:**

Parents of young children and clinicians at four urban pediatric practices participated in focus groups prior to implementation of screening. Participants were queried regarding attitudes, social norms, and barriers to developmental screening. Using information from the focus groups, workflow strategies were developed for implementing screening. Referral rates and satisfaction with screening were gathered at the conclusion.

**Results:**

Six focus groups of parents and clinicians were conducted. Major themes identified included 1) parents desired greater input on child development and increased time with physicians, 2) physicians did not fully trust parental input, 3) physicians preferred clinical acumen over screening tools, and 4) physicians lacked time and training to conduct screening. For the intervention, developmental screening was implemented at the 9-, 18-, 24-, and 30-month well visits using the Ages & Stages Questionnaire-II and the Modified Checklist for Toddlers. 1397 (98% of eligible) children under 36 months old were enrolled, and 1184 (84%) were screened at least once. 1002 parents (85%) completed a survey at the conclusion of the screening trial. Most parents reported no difficulty completing the screens (99%), felt the screens covered important areas of child development (98%), and felt they learned about their child’s strengths and limitations (88%).

**Conclusions:**

Developmental screening in urban low-income practices is feasible and acceptable, but requires strategies to capture parental input, provide training, facilitate referrals, and develop workflow procedures and electronic decision support.

## Background

The rates of detection of developmental delays are currently low. Approximately 12 to 16% of children are estimated to have developmental disorders [1,2]. However, only 30% of children with developmental delays are diagnosed before school entrance [3]. Low-income children are at greater risk for developmental delays, with increased rates of developmental delays reported in lower income children compared to higher income children [4]. More specifically, single-parent households and households in poverty have an increased rate of children with developmental problems [4,5]. Additionally, children with public health insurance are more likely to have special health care needs including developmental delays, and are at increased risk for long-term disability compared to children of higher socioeconomic status [6].

The identification of children with developmental delays before school entrance is vital to the well being of children. The adaptability of a child’s brain in the first 3 years of life makes identification of developmental delays and treatment with physical and psychosocial stimuli at a young age the foundation to a child’s developmental and behavioral outcomes [7,8]. Lower income children treated with early intervention programs from birth to age 5 years old have been shown to score significantly higher in reading and mathematics by age 15, as well as had fewer instances of grade retention and special education requirements compared to those children treated from ages 5 to 8 years old [9]. The readiness of children for school, especially low income children, may also help circumvent the consequences of early academic failure and school behavior problems including high school drop-out rates, delinquency, unemployment, and mental health issues in young adulthood [8].

Due to the need for diagnosing developmental delay early in a child’s life and improving detection rates, recommendations for developmental screening in young children were made in 2006 by the American Academy of Pediatrics (AAP) and the Maternal Child Health Bureau (MCHB) [10]. These recommendations encouraged primary care clinicians to provide developmental surveillance at all well child visits and institute developmental screening with validated tools at critical developmental periods in childhood (i.e., at 9, 18, and 30 months of age). An additional recommendation included autism-specific screening at the 18 and 24-month visits [11]. *Screening* was defined as the use of brief standardized tools that have relatively high sensitivity and specificity for the specific population’s risk status, are reliable, and focus on all developmental domains at specific age intervals to identify developmental delays [10]. *Surveillance* was defined as the process of recognizing children at risk for developmental delays through maintaining accurate documentation for the child’s developmental history in the child’s medical record, asking parents about their child’s development, and observing the child’s development in addition to the physical exam without the use of a standardized screening tool [10]. Surveillance was more closely defined as “unstructured surveillance,” which relies on clinical acumen as opposed to “structured surveillance” that screening experts define as use of periodic screening tools [12]. The AAP’s recommendation emerged from the growing concern that primary care physicians under-identify young children with developmental delays [13].

The AAP’s recommendation regarding screening was made with limited information regarding the feasibility or broad acceptance of this policy, especially in urban settings that may have the most at-risk children [14,15]. Previous studies have suggested that few clinicians have implemented developmental screening into their practices despite the dissemination of recommendations supporting their use [16-20]. It is currently estimated that nearly half of pediatricians do not routinely use developmental screening tools for children under the age of 36 months [21]. This limited implementation of screening is not surprising given studies that have shown that physicians prefer to rely on developmental surveillance rather than developmental screening. However the limited implementation of screening is problematic as developmental surveillance alone may identify fewer than half of children with developmental delays [22,23].

A growing body of literature suggests developmental screening is both effective and feasible if potential barriers are addressed adequately [24]. Barriers to screening that have been identified previously include lack of clinician knowledge and training, lack of adequate reimbursement for conducting screening, and the need to develop clinical workflow plans carefully [14]. In order to provide clinicians in urban low-income primary care settings with the information to undertake screening, we sought to contribute to the knowledge base by identifying challenges for developmental screening in these settings, developing strategies for conducting screening, and assessing the feasibility and acceptability of implementing a screening strategy. Specifically, we sought to employ a mixed methods study consisting of focus groups to identify the beliefs, practices, and perceived challenges that would contribute to poor adoption of developmental screening, and to use that information to inform implementation of developmental screening, rates of screening, and levels of satisfaction among parents and clinicians.

## Methods

### Setting

This mixed methods study was conducted from December 2008 to June 2010 at four urban pediatric primary care practices in Philadelphia. These practices experience more than 86,000 annual visits (22,500 under the age of five years). Children were eligible to participate if they were younger than 30 months old at the time of a visit, were greater than 36 weeks estimated gestational age, had no major congenital anomalies or genetic syndromes, were never placed in out-of-home foster care, and were not currently receiving Early Intervention Part C services. Clinicians were eligible if they were attending pediatricians, nurse practitioners, or pediatric residents at any of the participating practices. Medical students were excluded. The study was approved by the Institutional Review Board at the Children's Hospital of Philadelphia. Eligible participants including both parents and clinicians completed written informed consent. This study was a prelude to a clinical trial [25].

### Focus groups

Prior to implementation of screening, we conducted two focus groups with parents and four focus groups with clinicians to identify perceptions related to developmental screening. Parents of children under the age of 5 were recruited to participate, as well as clinicians at the four participating practices. We used the Theory of Planned Behavior as a conceptual framework to guide our inquiry [26,27]. This model proposes that the strength of an individual’s intentions to adopt a new behavior, developmental screening in this case, is dependent on their attitudes toward the behavior, the subjective norms and beliefs of those around them, and their perceived behavioral control (i.e., challenges they believed prevented them from conducting developmental screening) [26,27].

Each focus group meeting consisted of four to eight participants [28]. Meetings lasted for approximately one hour and were led by trained facilitators. Each meeting consisted of open-ended questions followed by a summary of responses. The questions sought to identify perceptions related to the importance of developmental screening, current screening practices, and challenges to implementing universal screening in order to more accurately identify these challenges for implementation of developmental screening. For example, one question used in the focus groups for pediatricians was, “In the care of young children less than five years old in primary care settings, how important is periodic screening of the child’s development compared with other aspects of well child care?” Similar questions were adapted for the parent focus groups. To improve the validity of our findings, we summarized the main ideas and sought participant feedback at the conclusion of each meeting. An investigator (J.G.) was present at all meetings to record field notes.

All focus group meetings were audiotaped and transcribed. Transcripts were entered into Ethnograph 6.0 (Qualis Research Associates, Thousand Oaks, CA), a qualitative software program that allows users to code, organize, and conduct searches for themes across transcripts. Field notes compiled at the meetings were used to supplement information from the transcripts. Transcripts were initially read by seven investigators (D.M., S.P., A.B., N.B., M.G., J.P., J.G.) to identify major themes. This team of investigators included a diverse array of individuals from general pediatrics, developmental-behavioral pediatrics, psychology, and qualitative research methods. Reliability in the selection of themes was ensured during an investigator meeting that achieved consensus on the overall themes and code lists. Based on this list of themes, investigators (D.M. and A.B.) independently reread and coded all transcripts. The themes were coded using the constant comparative approach, and any differences were settled by consensus [29].

### Implementation procedures

We developed an implementation strategy that encompassed the challenges identified in the focus groups including selection of developmental screening tools, clinician training methods, development of clinical workflow patterns, use of electronic decision support tools, and building a collaborative relationship with local early intervention (EI) agencies to share data. We garnered clinician input on the selection of screening tools through meetings at all four participating practices. After selection of tools, we developed an in-person training session to provide clinicians with an overview of the tools, information on administration and scoring of the tools, suggested text for interpreting positive screens with parents, and recommended referral procedures. Finally, we held meetings with staff from our local EI agency to develop agreements to share data on the status of EI referrals.

### Measures

Our quantitative outcome measures were screening rates and clinician and parent satisfaction with screening processes. We developed ad hoc measures of satisfaction regarding developmental screening, ease and use of tools, challenges to screening, and overall satisfaction with screening. For each item, satisfaction was rated on a five-point likert scale from 1 = very dissatisfied to 5 = very satisfied. Clinicians were e-mailed a link to an electronic version of the survey following the completion of the study period. In the email, they were thanked for participating in the study, but were not provided with any incentives for completion of the survey. Parents were called by study staff and completed a phone survey similar to the clinician survey. Parents were queried concerning demographic characteristics, knowledge and attitudes towards screening, ease and use of tools, outcomes of screening, and overall satisfaction. Clinician and parent satisfaction surveys were entered into a Research Electronic Data Capture (REDCap) database at the Children’s Hospital of Philadelphia [30].

We collected information from our electronic health records (EHR) on the number of patient well child visits, developmental screening and surveillance tools completed, and the number of EI referrals made during the study period. We obtained EI referral data from the participating EI agency and merged it with EHR data using personal identifying information (medical record numbers, child name, child date of birth). Once data were merged, all personal health information was deleted prior to analysis. Prior to the screening intervention, referral to EI was approximately less than 10%, but no exact data existed at the local EI agency or at the Children’s Hospital of Philadelphia for accurate comparison.

### Analysis

We completed summary statistics on parent and clinician satisfaction surveys as well as EHR and EI referral data. The parents were not the same parents that were involved in the focus groups, however many of the same clinicians participated in both the focus groups and the satisfaction questionnaires. We categorized parent and clinician responses of “satisfied,” “very satisfied,” or “yes” as indicating agreement with each statement. We aggregated the proportion of patients who had a well visit, completed a developmental screening tool, had an EI referral, and completed an EI referral that included a multidisciplinary evaluation during the study period.

## Results

Six focus groups (two parent and four clinician groups) were conducted, involving a total of 8 parents and 22 primary care clinicians. The parents that participated were female and predominantly African-American, reflecting the population from which they were drawn (Table 1). Clinician participants were mostly female and Caucasian, and had been in practice an average of 16 years (Table 1). All except one clinician were Board Certified in Pediatrics. Themes derived from the focus groups were categorized using the Theory of Planned Behavior [26,27]. We used three major categories to categorize themes according to the theoretical model: attitudes towards development, subjective norms towards screening, and perceived behavioral control towards implementing screening practices in the primary care setting.

**Table 1:** Characteristics of focus groups participants

**Characteristics**	**Parents**	**Clinicians**
	**N = 8**	**N = 22**
Mean age in years (SD)	33.4 (6.5)	42.9 (8.5)
Gender (%)		
Male	0 (0%)	1 (4.6%)
Female	8 (100%)	21 (95.4%)
Race/Ethnicity (%)		
White	2 (25%)	17 (77.3%)
African American	5 (62.5%)	2 (9.1%)
Hispanic	1 (12.5%)	0
Asian	0	3 (13.6%)
Education (%)		
High school graduate	5 (62.5%)	-
Some college	2 (25%)	-
More than college	1 (12.5%)	22 (100%)
Certified by American Board of Pediatrics	-	21 (95.4%)
Mean years of practice (SD)		16.1 (17.8)

### Attitudes towards development

Both parents and clinicians endorsed the importance of discussing the child’s development during the well visit. However, parents felt pediatricians undervalued parental knowledge and concerns about child development (Table 2). Parents voiced a desire to provide greater input on their child’s development and share in treatment decisions. Conversely, clinicians perceived that parents in their practice lacked knowledge of normal development. They reported that they routinely did not rely solely on parental report of development, but utilized a combination of parental report, clinician observation, and clinician expertise to assess development (Table 2).

**Table 2:** Perceived challenges to screening from focus groups

**Themes**	**Parents**	**Clinicians**
Parents desire greater input on child development, but clinicians do not trust parental knowledge of development.	*“…they (the Clinicians) [are] looking at their eyes and stuff, but you [are] never saying, ‘Mom, what do you see when you go home?’”*	*“…when I use the questions in EPIC [the electronic health record], if they say draw a circle, I don’t ask the parents, ‘Can they draw a circle?’ I actually have the child draw a circle or, you know, have the child hop. I have them do the tasks that are on there. And rarely the parent says, ‘Oh, they can do that,’ because a lot of times I'll have the parent say, ‘They can do that,’ and then the child can’t do that, you know? Often, the parents I have overestimate their [child’s] abilities*.”
Clinicians do not use validated screening tools, but rely on their clinical acumen and prefer to watch and wait.	*“…it comes back that she had a delay in reading. I've been complaining about it for so long; nobody would listen to me… We come in with questions like, ‘My child is fighting every day. My child is not being around… socializing. ’You know, and all you can - all they could say is, ‘Oh, give them a chance.’”*	Clinician 1: *“Most of us are just doing developmental surveillance. So we’re sort of looking; we’re not doing a full-on screening…”*
		Clinician 2: *“(When unsure about delay) I say, you know, ‘He’s not doing quite what we’d expect him to do. We’ll see how he’s doing in a couple of months…’”*
Well child visits as currently structured do not allow sufficient time, training, or resources to conduct developmental screening.	*“I do think that they’re all under heavy time constraints, and in getting people out the door as fast as possible, so there’s no time for conversation that may bring about certain issues.”*	*“…if it’s a tool that involves things like building blocks or crayons, it’s having them at your fingertips when you’re in the room and having access to them as well as time. So we do have kits. It involves 40 steps back that way and then 40 steps back the other way to get the kit and bring it into the room. If you kept them in the room, they would be taken home by the parents and the kids, so it’s about having what you need to fully do a tool.”*
		*“…it’s about having the components that you need to do the tool, and then knowing about the tool and how to do it properly.”*

### Subjective norms towards screening

In these urban practices prior to this study, clinicians acknowledged that they frequently employed surveillance to assess development, relying on a set of age-specific milestones that were incorporated into the electronic health record to identify delays at well child visits. Clinicians reported validated developmental screening tools were used sporadically and only when parents raised concerns (Table 2). Likewise, parents felt clinicians did not spend adequate time to assess their children’s development. They desired more information on development for their children (Table 2). Parents preferred clinicians to be more proactive in referring their children to developmental services rather than using a watch-and-wait approach for screening and referral.

### Perceived behavioral control toward implementing screening practices

Clinicians identified the following challenges to screening: lack of time to conduct screening, lack of reimbursement for completing developmental screening tools, and lack of training in the use of developmental screening tools. Time constraints within busy well child visits were recognized, and clinicians perceived that they could not eliminate important aspects of well childcare to accommodate screening (Table 2). At the time of this study, developmental screening was not reimbursed in these four practices. Clinicians acknowledged that additional funding might help facilitate screening by paying clinic staff to assist with screening procedures or allotting extra time during well visits to conduct screening. Finally, clinicians perceived a lack of training on the administration, scoring, and interpretation of tools.

### Strategy for implementing screening

Using the information gathered from the focus groups, namely parents’ desire for more input on development and clinicians’ preference for a brief, validated, and global developmental screening tool, we made a list of validated screening tools for consideration in this urban clinic setting (Table 3). Table 3 illustrates our implementation of developmental screening strategy that includes suggestions from our focus groups. We convened an additional meeting with clinicians from participating practices in order to review the AAP recommendations for developmental screening and to provide an overview of a number of tools. Based on feedback from that meeting, we selected the Ages & Stages Questionnaires, Second Edition (ASQ-II) as a general developmental screener at the 9-, 18-, and 30-month well visits [31]. The ASQ-II accommodated parental self-report, clinicians’ preference for a milestone-based instrument, and adequate speech and language assessment that could also provide an educational tool for house staff, residents, and parents (Table 3). It was also recognized that the local EI agency used the ASQ-II as a first-stage screening tool for the evaluation of children with possible delays. We also selected the Modified Checklist for Autism in Toddlers (M-CHAT) at the 18- and 24-month well visits as it was a similarly validated parent self-report tool and was the only brief autism screener available in this age group [32].

**Table 3:** Implementation strategy for developmental screening

**Strategy domain**	**Goals to screening**	**Decision and action**
1. Selection of developmental screening tools	A. To include parents’ desire for input: can be concerns-based or milestone-based reporting	I. Ages & Stages Questionnaires, Second Edition
		i. 9, 18, and 30 month visits
		ii. Parents given tool on paper at check-in
	B. To include clinicians’ preference for a brief, validated, global developmental screening tool with multiple milestone domains	
		iii. Clinician scores tool at visit
		II. Modified Checklist for Autism in Toddlers (M-CHAT)
		i. 18 and 24 month visits
		ii. Parent given tool on paper at check-in
		iii. Scored by clinician at visit
2. Training & education	A. To provide incentives for completing training	I. Developed training video
	B. To have clinic staff provide reinforcement for training	II. Both group and individual training at clinician discretion
	C. To give a flexible format for training	III. Provided CME credit
		IV. Incorporated resident training on developmental tools into overall residency curriculum
		V. On-site clinic staff to answer questions and provide guidance
3. Electronic clinical decision support tools to sustain screening	A. To utilize electronic decision support for automated scoring and identification of subjects for speed and readiness	I. Placement of PDF of ASQ-II in the EHR with live scoring grid that automatically calculates score
		II. Provide M-CHAT questions in electronic format with live scoring grid that automatically calculates score
		III. Screening reminder alerts for 9-, 18-, 24-, and 30-month well child visits
		IV. Electronic EI health appraisals and prescriptions to facilitate faxing of referrals
4. Develop workflow procedures	A. To develop a feasible and efficient workflow to implement screening at designated well-child visits	I. Mail reminder letters 45 days prior to scheduled study visits
		II. Mail questionnaires 15 days before appointment date
	B. To utilize clinic staff to help facilitate workflow procedures	III. Automated reminder phone call 1 day before visit
		IV. Screening tools prepared with clipboards 1 day before visit; given upon arrival at check-in
		V. Administer/score tools and enter results in electronic health record prior to clinician visit
		VI. Clinician interprets scores and provides feedback to family; clinician completes well-child visit, makes decision to refer, and faxes EI forms to EI
5. Facilitate referrals & data	A. To collaborate with Early Intervention to track referrals and follow-up	I. Agreement with EI to share data and allow faxing of EI health appraisal/prescriptions
		II. Quarterly tracking spreadsheet generated and maintained by each practice and updated by EI
		III. Agreement with EI to accept ASQ-II/M-CHAT results from screening as part of intake
		IV. Determination of child’s EI status

We provided training and education of Pediatric Residents and Attendings at their discretion in order to meet the clinicians’ desires from the focus groups of providing sufficient training and resources on developmental screening (Table 3). Group meetings were conveniently scheduled at each participating practice to conduct clinician training. At these meetings, we reviewed developmental screening recommendations, provided an overview of the screening tools selected, and gave hands-on instruction in the administration, scoring, and interpretation of the tools. For those who could not attend a group training, a training video was developed that could be reviewed at their leisure. Continuing medical education credits were provided to Attendings as an incentive to complete training, and the developmental screening tools were incorporated into the overall residency curriculum. For additional assistance, on-site clinic staff was available in each clinic to assist clinicians with screening.

We developed and incorporated electronic clinical decision support tools to support developmental screening at recommended well visits in order to include the clinicians’ request to have better access to developmental screening resources (Table 3). Alerts were automatically generated to remind clinicians that screening was due at a particular well visit. For the ASQ-II, an age-specific portable document format (PDF) was available through a link within the EHR that could be printed and provided to parents to complete. Clinicians could transfer parent responses to the EHR by selecting the appropriate radio buttons conforming to responses on a scoring grid, and an automated scoring algorithm would tally the responses and provide an overall score to minimize errors. For the M-CHAT, an electronic interactive version of the questions was available within the EHR. Similarly, clinicians could transfer parent responses to an automated scoring grid, which would provide an overall score.

**Figure 1:**
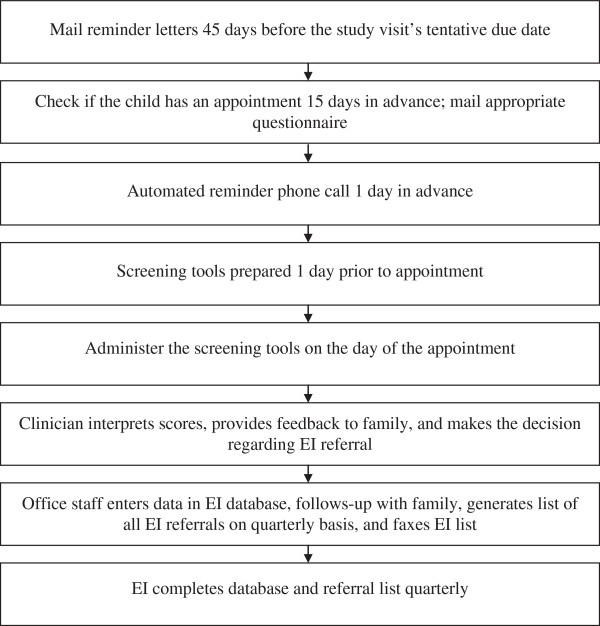
Workflow procedures.

To facilitate implementation and improve clinician efficiency as identified in the focus groups, we developed a workflow procedure for the implementation of screening (Figure 1, Table 3). According to the workflow, 15 days prior to scheduled well visits, parents were mailed the age-appropriate screening tools with instructions to complete the tools and bring them to their child’s upcoming well visit. Automated phone calls were made one day prior to scheduled visits to remind parents of the visit and to complete the screening tools. On the day of the visit, front desk staff took completed screeners from parents at check-in. If parents did not bring their pre-mailed screening tool completed, office staff provided parents with available screeners. Parents completed or had assistance from office staff to complete screening tools. Completed screening tools were either provided to clinicians or results were entered into the EHR by office staff prior to clinicians seeing the patients.

We developed a letter of agreement with the county EI agency preceding the study to permit information on referral status to be faxed to EI agency staff (Table 3). The clinic staff maintained a monthly tracking spreadsheet that early intervention agencies would update on a quarterly basis. This spreadsheet included information on the child’s date of assessment, referral date, medical record number, age, the status of enrollment in EI services, and the scheduling and evaluation results of multidisciplinary evaluations (MDE). The EI agency agreed to accept the ASQ-II/M-CHAT results as part of their intake for developmental evaluations. EI staff reported on the status of all referrals: referral intake, completion of referrals, scheduling of MDE, and results of MDE and status of services.

### Screening results

One thousand three hundred ninety-seven eligible children under 31 months old were enrolled and followed for up to 18 months, and 1,184 (84.8%) parents/caregivers completed a developmental screening tool at least once during the study period (Table 4). Most children were male, African American, had mean family incomes of less than $30,000, and had a parent with greater than a high school education (see Additional file 1). There were no differences (p > 0.05) in demographic characteristics by practice site. Comparing the focus group caregiver participants to the screening intervention participants, the screening intervention parents were of slightly higher education levels, but of similar race and ethnicity (Table 1 and Additional file 1). Developmental screening resulted in 348 (24.9%) children being identified with developmental delays (Table 4). Two hundred fifty one children (18.0%) were referred for EI services, and 128 (9.2%) completed an EI referral (Table 4).

**Table 4:** Results of developmental screening

**Outcome**	**Eligible children**
	**N = 1397**
Number attended Well Visit (%)	1363 (97.6%)
Number screened at Visits (%)	1184 (84.8%)
Number identified with Delays (%)	348 (24.9%)
Number referred to Early Intervention (%)	251 (18.0%)
Number completed Early Intervention referrals (%)	128 (9.2%)

Once a parent completed a developmental screening visit, parents and clinicians were asked to complete satisfaction questionnaires, which typically occurred between one and 12 months after the well-child visit. Of the 1,184 who completed a developmental screen at a well visit and were eligible to participate in the survey, 1,016 parents (85.8%) completed the phone survey at the conclusion of the study. Most parents reported no difficulty completing the screens (98.6%), the screens covered important areas of child development (97.6%), and that the developmental screening tools helped them learn about their child’s strengths and challenges (88.3%) (Table 5). Of the 208 Attendings, Nurse Practitioners, and Residents, 123 (59.1%) completed the on-line survey. One-hundred sixteen clinicians (94.3%) felt that developmental assessment was an important part of well-child care (Table 6). Only 67 (54.5%) felt caregivers have a good understanding of typical child development. However, 120 (97.6%) valued the importance of seeking parental input regarding child development. Overall, most parents (98.5%) and clinicians (70.8%) reported satisfaction with developmental screening.

**Table 5:** Caregiver satisfaction with screening

**Items**	**Parent response**	
	**N = 1016**	
I am satisfied with answering questions on development at the well-child visit	Agree	1002 (98.6%)
The developmental tool is understandable	Agree	1006 (99.3%)
The developmental tool covers all important areas of development	Agree	978 (97.6%)
The developmental tool helps parents understand their child’s developmental strengths and challenges	Agree	893 (88.3%)
Parents learned of activities to help their child grow and learn during the well-child visit	Agree	780 (82.0%)
Parents had additional concerns or questions that needed more attention than the child’s development	Disagree	962 (95.2%)
I am satisfied with my child’s developmental assessment	Agree	513 (98.5%)

**Table 6:** Clinician satisfaction with screening

**Items**	**Clinician response**	
	**N = 123**	
Assessment of development is an important part of well-child care	Agree	116 (94.3%)
Caregivers have a good understanding of typical child development	Agree	67 (54.5%)
It is important to seek caregiver input regard their children’s development	Agree	120 (97.6%)
The ASQ-II or M-CHAT is easy for parents/caregivers to complete	Agree	74 (71.2%)
The ASQ-II or M-CHAT is easy to score in EHR	Agree	75 (82.4%)
The ASQ-II or M-CHAT is quick to complete	Agree	59 (55.7%)
The ASQ-II & M-CHAT are helpful in my clinical decision-making	Agree	92 (84.4%)
Developmental screening (with the ASQ-II/M-CHAT) disrupts my clinical workflow	Agree	46 (42.2%)
I have received sufficient training on how to administer the ASQ-II/M-CHAT	Agree	61 (56.0%)
The clinic staff provides helpful developmental support to families	Agree	108 (93.9%)
The clinic staff is helpful with Early Intervention referral and tracking	Agree	92 (82.9%)
I am satisfied with the developmental screening process (i.e. using the ASQ-II and M-CHAT) at my clinic	Agree	85 (70.8%)

## Discussion

Many of our findings are consistent with other research, including our focus group finding that parents desired a greater input on developmental decisions [33]. Prior research with parents of developmentally delayed children found that parents raised concerns about their child’s development more than a year before clinicians recognized a problem [34,35]. These and other studies document that raising simple questions of concern with parents about a child’s development and learning may yield important information leading to identification of a problem [10,36], have a positive impact on timely diagnoses of delays in young children [36-39], and increase referral rates in developmentally delayed children as opposed to clinicians using a watch-and-wait approach to referring children to early intervention programs [14,39,40]. Thus, we sought to incorporate parent-report measures in our selection of appropriate screening tools.

Clinicians in this study perceived challenges to developmental screening including insufficient time and lack of training on developmental screening tools that have been noted in earlier reports [7,41]. A study on two urban primary care practices found routine screening to be more feasible than expected when they addressed issues of time and training as we did in our screening strategy [24]. Clinicians in our study also identified workflow plans as an important factor in efficiently incorporating developmental screening, which has been found in other studies [24,42]. When clinicians were deciding on an appropriate screening tool for their urban practices, the ASQ-II and M-CHAT were selected based on concurrent use by the local early intervention agency, their basis in milestones and parent report, and their ability to reinforce teaching on child development to residents and parents. Prior research has similarly shown decisions regarding screening tools are based on clinical flow, acceptance by local outreach programs or early intervention, and their ability to teach typical child development [42]. By addressing these challenges and implementing screening within current workflow parameters, participating practices showed a high rate of screening (84.8%). This is comparable to findings from other studies that have implemented screening strategies [42], but higher than that achieved by other urban clinics that have implemented screening [24].

Despite our high rate of screening, only 9.2% of children completed referrals to Early Intervention. Three-hundred forty eight children (24.9%) were identified with developmental delays through screening, but only 251 (65.4%) of those children were referred to Early Intervention. This may imply a higher reliance by pediatricians on clinical acumen and structured surveillance than on developmental screening tools, but more study is needed to assess this assertion. A greater proportion of those referred were male (p < 0.0001) or of African American race (p < 0.001). Of those referred to Early Intervention, 128 (51%) completed the referral. We do not fully understand why only half of parents completed the referral to Early Intervention with their child. We speculate that barriers to referral completion may be present. For example, in the satisfaction questionnaires given to parents after the implementation of screening, 236 parents answered, “Yes” to their child being recommended for Early Intervention. However, of those 236, only 189 (80%) agreed with the recommendation for referral. In addition there may be other factors that limit this urban population from completing referrals including lack of knowledge on the need for seeking Early Intervention services, lack of time, and limited resources such as disconnected phone numbers. In a study by Garg et al. that researched the impact of a family help desk at an urban clinic for low-income children, a disparity existed between the initial contact of parents and their receipt of community resources [43]. The barriers faced by low-income families including time constraints, childcare, and transportation issues were mentioned as possible reasons for this disconnect, but more research is needed on the subject [43]. Although a greater proportion of referred children were male or of African American race, we could not identify children for screening based on demographic characteristics. Moreover, our results show that once a clinician makes an EI referral in this high-risk population, additional care coordinator resources may be needed to facilitate the referrals [25].

Limitations to our findings exist. First, this study was conducted in a single geographical area using a non-random sample of participants from four practices. Thus, our findings may not be generalized to other geographic areas or other practices in the same geographic area. Second, our utilization of clinical work staff for dispersing screening tools to parents, screening children, entering screening tool results into the EHR, and keeping a quarterly-updated spreadsheet for EI referrals and correspondence on follow-up may not be feasible at other clinics. However, many of the patients participating in our study did not avail themselves of assistance from clinic staff and were still able to complete developmental screening [42]. Third, parents and clinicians were given satisfaction questionnaires between one and 12 months after their screening visit, which may have resulted in recall issues in answering satisfaction questions about the developmental screening. However, few parents and clinicians reported that they were unable to recall screening.

Despite these limitations, we believe that our findings have valuable implications for pediatric practices. Our results show that developmental screening is feasible in a high-risk, low-income population. By utilizing the input of parents and providers from urban primary care practice, we were able to create a workflow for screening that fit our practices, and received feedback in the form of satisfaction surveys to establish the acceptability of our strategy. Our results also suggest that clinicians in urban settings can utilize parental report from developmental screening tools to screen for developmental delays, provided sufficient practice-based resources are available such as clinician training and point-of-care electronic reminders. Other studies have found referral-tracking efforts to be too labor-intensive for clinic staff [42]. However, we have found that forming an agreement with an early intervention agency to share referral data and maintaining a referral spreadsheet by clinic staff was a successful way to track referrals.

The widespread adoption of clinical workflow procedures for implementing developmental screening has the potential to lead to greater identification of developmental delays in young children. Children of low socioeconomic status are at increased risk for developmental delays, and the adoption of effective and efficient developmental screening strategies can improve identification of delay in high-risk populations. Early developmental screening is an important strategy for identifying and helping children with delays as recommended by the AAP. This is especially true in urban clinics that serve a predominantly low socioeconomic population.

## Conclusions

In this mixed methods study, parents and clinicians perceived developmental screening favorably, but a number of challenges to screening were identified. These included lack of agreement on whether parents could give accurate assessments of child development, clinician preference to rely on their clinical acumen, and limited time, insurance reimbursement, and training on screening. With knowledge of these perceived challenges, we utilized clinician input to select parent-reported screening tools, developed workflow procedures to enhance screening efficiency, provided clinician training using flexible formats, implemented electronic decision tools to support screening, and made collaborative arrangements with EI agencies to share data on the results of screening and referrals. These strategies resulted in 84.8% of children being successfully screened. In addition, parents and clinicians reported overall satisfaction with screening procedures.

## Abbreviations

AAP: American academy of pediatrics; ASQ-II: Ages & stages questionnaires, second edition; CME: Continuing medical education; EHR: Electronic health record; EI: Early intervention; M-CHAT: Modified checklist for autism in toddlers; MDE: Multidisciplinary evaluations; PDF: Portable document format.

## Competing interests

No competing interests have been identified with any of the authors.

## Authors’ contributions

JG conceived of the study, wrote the grant and protocol, acquired the data, analyzed and interpreted the data, and drafted and revised the manuscript. DM helped conduct the study, interpreted and analyzed the data, and drafted and revised the manuscript. SP helped critically revise the manuscript and interpret the data. AB helped conduct the study, interpret and analyze the data, and critically revise the manuscript. NJB helped critically revise the manuscript and interpret data. MG helped conceive of the study, conduct the study, and critically revise the manuscript. JPM critically revised the manuscript and analysis. All authors read and approved the final manuscript.

## Pre-publication history

The pre-publication history for this paper can be accessed here:

http://www.biomedcentral.com/1471-2431/14/16/prepub

## Supplementary Material

Additional file 1 **Characteristics of children screened.** A demographic comparison of children who were screened for developmental delay, identified with delay, referred to early intervention, and completed the early intervention referral. P-values were reported to show significance. Click here for file
