# Supplementary material for: Challenges to implementation of developmental screening in urban primary care: a mixed methods study
Source: BMC Pediatr. 2014 Jan 21;14:16. doi: 10.1186/1471-2431-14-16 (PMC3899611; doi:10.1186/1471-2431-14-16)
Supplement: Additional file 1 — Characteristics of children screened. A demographic comparison of children who were screened for developmental delay, identified with delay, referred to early intervention, and completed the early intervention referral. P-values were reported to show significance. [file 1471-2431-14-16-S1.docx]

| **Characteristics** | **Total Children Screened** | **Attended Well Visit** | | **Screened** | | | **Identified With Delay** | | **EI Referral** | | | **EI Completion** | |
| --- | --- | --- | --- | --- | --- | --- | --- | --- | --- | --- | --- | --- | --- |
|  | (N=1397) | (n=1363) | *P** | (n=1184) | *P* | | (n=348) | *P* | (n=251) | | *P* | (n=128) | *P* |
| Age at enrollment (month) |  |  |  |  |  | |  |  |  | |  |  |  |
| Mean (SD) | 11.1 (8.3) | 10.8 (8.1) | <.0001 | 10.8 (8.0) | 0.25 | | 11.4 (7.2) | 0.04 | 12.3 (7.0) | | <0.001 | 11.5 (6.4) | 0.1 |
| Sex (%) |  |  | 0.49 |  | 0.05 | |  | <.0001 |  | | <.0001 |  | <.0001 |
| Male | 705 (50.5) | 690 (50.6) |  | 590 (49.8) |  | | 215 (61.8) |  | 159 (63.3) | |  | 88 (68.8) |  |
| Female | 692 (49.5) | 673 (49.4) |  | 594 (50.2) |  | | 133 (38.2) |  | 92 (36.7) | |  | 40 (31.2) |  |
| Race (%) |  |  | 0.79 |  | 0.03 | |  | 0.03 |  | | <0.001 |  | 0.43 |
| African American | 1071 (76.7) | 1046 (76.7) |  | 917 (77.5) |  | | 284 (81.6) |  | 214 (85.3) | |  | 103 (80.5) |  |
| Caucasian | 171 (12.2) | 166 (12.2) |  | 147 (12.4) |  | | 32 (9.2) |  | 15 (6.0) | |  | 11 (8.6) |  |
| Other | 155 (11.1) | 151 (11.1) |  | 120 (10.1) |  | | 32 (9.2) |  | 22 (8.8) | |  | 14 (10.9) |  |
| Ethnicity (%) |  |  | 1** |  | 0.48 | |  | 0.56 |  | | 0.66 |  | 0.57 |
| Hispanic | 36 (2.6) | 36 (2.6) |  | 29 (2.5) |  | | 7 (2.0) |  | 5 (2.0) | |  | 4 (3.1) |  |
| Non-Hispanic or others | 1361 (97.42) | 1327 (97.4) |  | 1155 (97.5) |  | | 341 (98) |  | 246 (98) | |  | 124 (96.9) |  |
| Family Income (%) |  |  | 0.12 |  | 0.63 | |  | 0.13 |  | | 0.3 |  | 0.72 |
| Missing | 7 (0.5) | 6 (0.4) |  | 6 (0.5) |  | | 4 (1.1) |  | 1 (0.4) | |  | 1 (0.78) |  |
| <$30,000 | 861 (61.6) | 838 (61.8) |  | 724 (61.2) |  | | 217 (62.4) |  | 165 (65.7) | |  | 79 (61.7) |  |
| >=$30,000 | 529 (37.9) | 519 (38.1) |  | 454 (38.3) |  | | 127 (36.5) |  | 85 (33.9) | |  | 48 (37.5) |  |
| Care Taker Education (%) |  |  | 0.04 |  | 0.09 | |  | 0.04 |  | | 0.84 |  | 0.57 |
| Missing | 7 (0.5) | 6 (0.4) |  | 6 (0.51) |  | | 4 (1.2) |  | 1 (0.4) | |  | 1 (0.8) |  |
| <=High School | 689 (49.3) | 668 (49.0) |  | 570 (48.1) |  | | 158 (45.4) |  | 128 (51.0) | |  | 65 (50.8) |  |
| >High School | 701(50.2) | 689 (50.6) |  | 608 (51.3) |  | | 186 (53.4) |  | 122 (48.6) | |  | 62 (48.4) |  |
| * All p values are based on Fisher Exact Test except for those for age, which are based on Wilcoxon test Z score. | | | | | | | | | | |  |  |  |
| **25% of the cells have expected counts less than 5. | | | |  | |  |  |  | |  |  |  |  |
